# Influence of intrinsic and synaptic properties on transmission of spike timing precision

**DOI:** 10.1186/1471-2202-14-S1-P31

**Published:** 2013-07-08

**Authors:** Heather A Brooks, Alla Borisyuk

**Affiliations:** 1 Department of Mathematics, University of Utah, Salt Lake City, UT 84112, USA

## 

Neuronal firing is intrinsically noisy, and as signals travel through nervous systems, the precision and reliability of the timing information is varied. At some processing stages spike timing precision is reduced through propagation; e.g., as in auditory signals transmitted from the brainstem to the inferior colliculus. On the other hand, timing information can be sharpened through propagation, as in transmission from the auditory nerve to the brainstem. Indeed, accurate coincidence detection is possible despite the inherent noise of the individual neurons. Motivated by this variability of timing information processing in the auditory system, we investigate how changes in the cellular and synaptic properties contribute to improved or decreased precision of spike timing through synaptic transmission.

We use a three-dimensional conductance-based model based on Morris-Lecar equations with added voltage-sensitive potassium M-current. Parameters are tuned to represent dynamics of hippocampal pyramidal cells [1]. The single neuron model receives an incoming spike through each of a number of input lines, with spike time drawn independently from a Normal distribution. We examine the influence of four synaptic properties (number of input lines, strength of input conductance, synaptic time constant, and standard deviation of input times) and two intrinsic states (integrator and coincidence detector; the model under consideration can be switched from one to the other through modulation of the leak conductance). In fact, the spiking behavior and spiking precision varies across this parameter space in a complex manner.

We identify regions in parameter space where the spike timing precision can be explained by the distributions of order statistics of input times. In this region, precision is mostly independent of synaptic time constant and the input strength, provided the input is strong enough. Rather, it is most strongly affected by the number of incoming input lines. This dependence is similar for both coincidence detector and integrator models. In other parameter regimes, the spiking precision depends more subtly on details of interaction between the adaptation mechanism (in the form of M-current) and leak (or background) conductance. We use phase plane and bifurcation analysis to explain results of our numerical observations.
